# Navigating Complexity: Transcatheter Aortic Valve Replacement (TAVR) in an Elderly Patient With Critical Aortic Stenosis and Severe Annular Calcification

**DOI:** 10.7759/cureus.93403

**Published:** 2025-09-28

**Authors:** Nouraldeen Manasrah, Dania Al Jabri, Sarah Alqasem, Ahmed Abdelrahman, Musa Sharkawi

**Affiliations:** 1 Cardiology, Augusta University Medical College of Georgia, Augusta, USA; 2 Internal Medicine, Augusta University Medical College of Georgia, Augusta, USA; 3 Internal Medicine, Luzmila Hospital, Amman, JOR; 4 Cardiology, Mount Sinai Medical Center, Miami, USA

**Keywords:** aortic valve disease, calcified aortic valve, paravalvular leak (pvl), percutaneous pvl (paravalvular leak) closure, trans catheter aortic valve implantation (tavi)

## Abstract

We present the case of a 93-year-old female patient with critical calcific aortic stenosis, severe annular and left ventricular outflow tract (LVOT) calcification, and reduced ejection fraction who underwent transcatheter aortic valve replacement (TAVR) with a 23-mm Edwards Sapien 3 Ultra valve. Post-procedure, she experienced a mild to moderate paravalvular leak (PVL) with an improvement in ejection fraction. At the one-month follow-up, worsening dyspnea and moderate to severe PVL were noted. Percutaneous PVL closure was successfully performed using an 8-mm Amplatzer Vascular Plug II, effectively reducing the leak to a trace. The patient was discharged the following day and remains independent with minimal symptoms at eight months. This case highlights the challenges of managing severe annular calcification during TAVR and the utility of PVL closure to improve clinical outcomes.

## Introduction

Transcatheter aortic valve replacement (TAVR) has emerged as a pivotal intervention for patients with severe calcific aortic stenosis, particularly for patients at high or prohibitive risk for surgical aortic valve replacement [1]. TAVR has demonstrated significant benefits in improving symptoms, functional status, and survival rates among high-risk patients with severe aortic stenosis [2].

Severe aortic annular and left ventricular outflow tract (LVOT) calcification poses a challenge when considering TAVR and is more pronounced in patients in advanced disease stages and with bicuspid aortic valves. Among patients undergoing TAVR, aortic valve and annular calcification has been identified as a predictor of paravalvular regurgitation and annular rupture [3].

The following case details TAVR in a 93-year-old female with critical calcific aortic stenosis with severe annular calcification, highlighting the complex decision-making process required to achieve optimal clinical outcomes.

## Case presentation

A 93-year-old female with a history of hypertension and stage 3A chronic kidney disease was transferred to our facility for management of new-onset acute heart failure with reduced ejection fraction and critical aortic stenosis. She had reported shortness of breath for at least five months but maintained independence in her daily activities. On presentation, vital signs were as follows: blood pressure 138/85 mmHg, heart rate 78 beats per minute, respiratory rate 16 breaths per minute, temperature 37.1°C, and oxygen saturation 96% on room air. The complete blood count and basic metabolic panel results were within normal limits, except for an elevated creatinine level of 1.7 mg/dL, which is her baseline, as summarized in Table 1.

**Table 1:** Complete blood count and basic metabolic panel laboratory results

Parameters	Normal Range	Patient’s Values
Complete Blood Count (CBC) Labs		
Hemoglobin (Hgb)	13.5–17.5 g/dL (men)	13 g/dL
	12.0–15.5 g/dL (women)	
Hematocrit (Hct)	38.8%–50% (men)	42%
	34.9%–44.5% (women)	
White Blood Cells (WBC)	4,500–11,000/µL	7,200/µL
Platelets	150,000–450,000/µL	285,000/µL
Mean Corpuscular Volume (MCV)	80–100 fL	90 fL
Basic Metabolic Panel (BMP) Labs		
Sodium (Na)	135–145 mmol/L	136 mmol/L
Potassium (K)	3.5–5.0 mmol/L	4.7 mmol/L
Chloride (Cl)	98–106 mmol/L	102 mmol/L
Bicarbonate (HCO_3_)	22–28 mmol/L	24 mmol/L
Blood Urea Nitrogen (BUN)	7–20 mg/dL	20 mg/dL
Creatinine (Cr)	0.6–1.3 mg/dL	1.7 mg/dL
Glucose	70–99 mg/dL (fasting)	105 mg/dL

**Figure 1:**
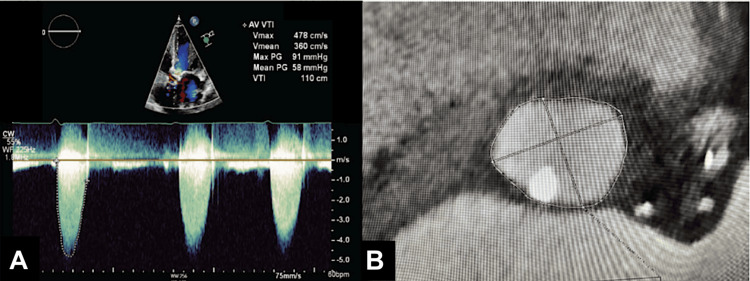
(A) Transthoracic echo shows severe aortic stenosis. (B) Prominent calcific nodule extending into the annulus and left ventricular outflow tract (LVOT) with aortic annular area measured at 418 mm². Figures reprinted from [12], ©2025 HMP Global. Used with permission.

Transthoracic echocardiography (TTE) revealed an ejection fraction of 20%-25% and critically severe aortic stenosis (aortic valve area of 0.26 cm², mean pressure gradient of 58 mmHg, dimensionless index of 0.16, and peak aortic velocity of 4.7 m/s) (Figure 1A). Right and left heart catheterization indicated preserved cardiac output and mild coronary artery disease. Computed tomography (CT) angiography showed peripheral vasculature suitable for a femoral approach for TAVR, as well as severe calcification of the aortic valve with a calcium score of 5478. Furthermore, there was a prominent calcific nodule extending into the annulus and LVOT, and the aortic annular area measured at 418 mm² (Figure 1B).

**Figure 2:**
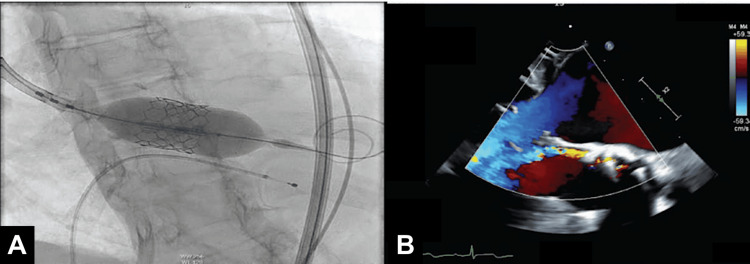
(A) A 23 mm Edwards Sapien 3 Ultra Resilia valve deployed. (B) A transesophageal echocardiogram (TEE) reveals a moderate to severe aortic paravalvular leak (PVL) following transcatheter aortic valve replacement (TAVR). Figures reprinted from [12], ©2025 HMP Global. Used with permission.

After a brief period of medical optimization, the patient underwent TAVR with a 23 mm Edwards Sapien 3 Ultra Resilia valve and cautious post-dilation with nominal balloon preparation (Figure 2A). Post-TAVR, her ejection fraction improved to 35%-40%, with a mean gradient of 11 mmHg and a mild to moderate paravalvular leak (PVL). The patient was discharged the following day with a plan for close follow-up.

At the one-month follow-up appointment, she reported recurrence of shortness of breath with activity. A repeat echocardiogram showed moderate to severe PVL, and her ejection fraction had improved to 40%-45%, with an aortic valve gradient of 9 mmHg. We elected to perform PVL closure. Intraoperative transesophageal echocardiography (TEE) revealed moderate to severe PVL, with two jets on either side of the large LVOT nodule (Figure 2B). Cardiac catheterization revealed a left ventricular end-diastolic pressure (LVEDP) of 24 mmHg and an aortic regurgitation index of 16.

Procedure details

**Figure 3:**
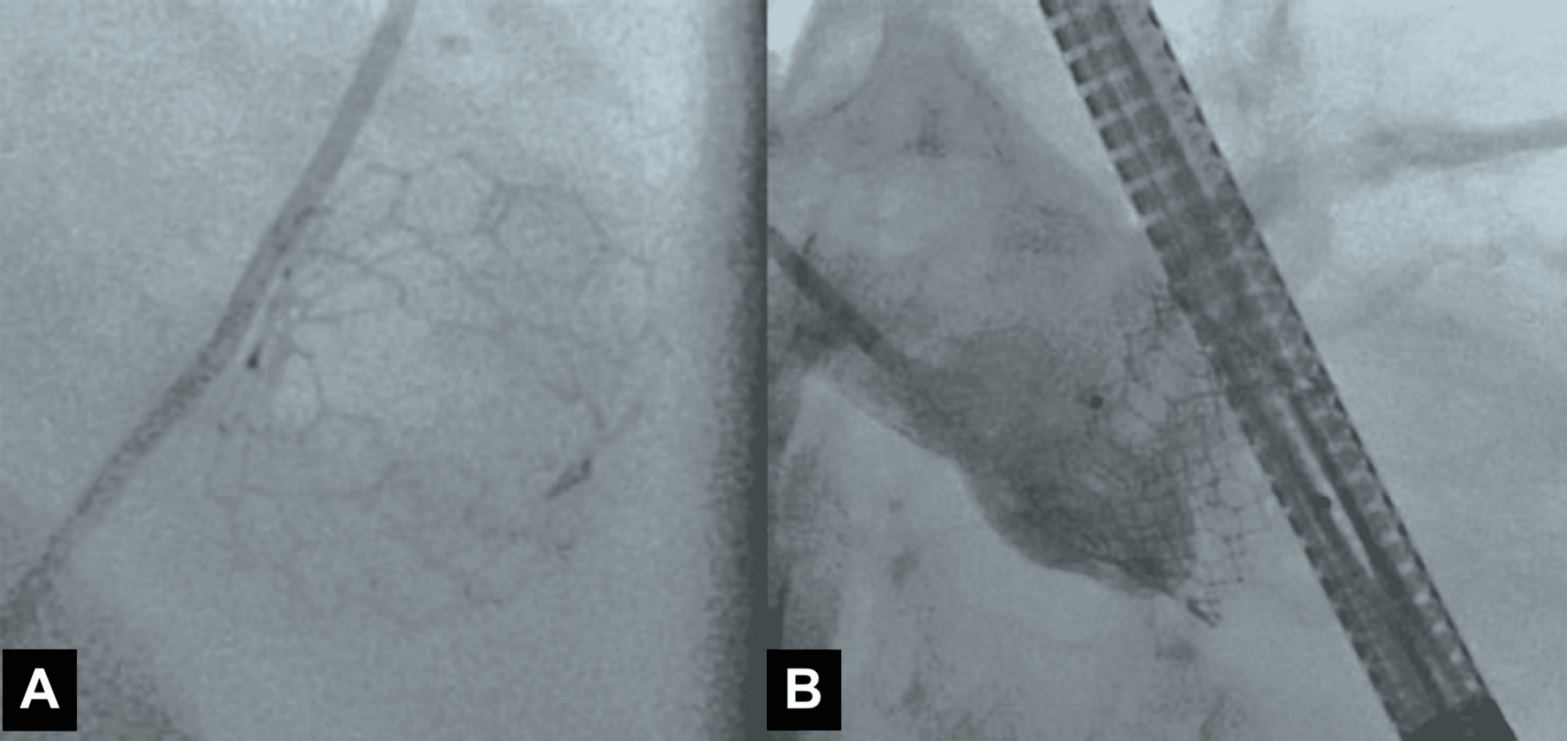
(A) Berenstein catheter advanced into the left ventricle and confirmed with fluoroscopy to be outside the valve frame. (B) Amplatzer Vascular Plug II (Abbott, Chicago, IL, USA) deployed with trace paravalvular leak (PVL). Figures reprinted from [12], ©2025 HMP Global. Used with permission.

A 7 French (Fr), 45 cm sheath was inserted via the right femoral artery. The PVL was crossed with a 7 Fr multipurpose guide catheter, a Berenstein catheter in a mother-daughter technique, and a Minamo coronary wire (Asahi Intecc, Seto, Japan). The Berenstein catheter was then advanced into the left ventricle and confirmed with fluoroscopy to be completely outside the valve frame (Figure 3A).

**Figure 4:**
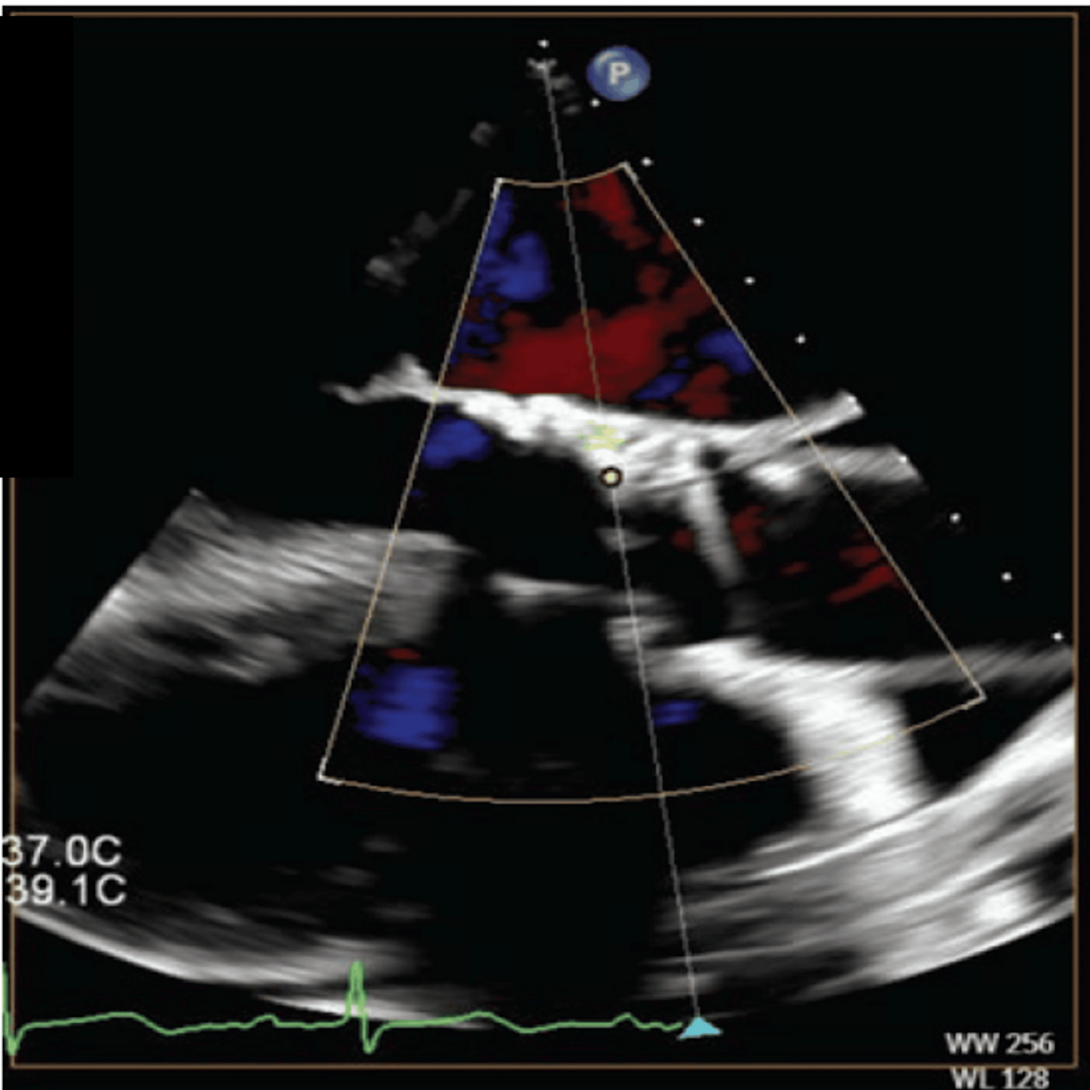
Transesophageal echocardiography (TEE) showing no paravalvular leak after successful deployment of the Amplatzer Vascular Plug. Figures reprinted from [12], ©2025 HMP Global. Used with permission.

A Confida wire (Medtronic, Dublin, Ireland) was placed in the left ventricular apex, after which the Berenstein catheter was removed. A 7 Fr Railway sheathless access system (Cordis Corp., Hialeah, FL, USA) was utilized to assist the multipurpose guide in crossing into the left ventricle, followed by deployment of an 8 mm Amplatzer Vascular Plug II (Abbott, Chicago, IL, USA) (Figure 3B). This was selected to ensure coverage of the two jets on either side of the calcific annular nodule with the proximal and distal disks. The plug was successfully deployed, reducing the PVL to a trace level. Intraoperative TEE showed trace PVL (Figure 4).

The patient was discharged on postoperative day one and remains fully independent, with minimal dyspnea, at the eight-month follow-up.

## Discussion

Calcification, especially in the form of eruptive nodules in the aortic annulus and LVOT, significantly increases the risk of complications of TAVR. Recent studies confirm a link between annular calcification and post-procedural aortic regurgitation, with findings from 177 patients indicating that the location and severity of calcification, especially in the LVOT, is a critical predictor of aortic regurgitation [3-5]. Notably, moderate to severe calcification in the LVOT is associated with a 2.5- to 5.4-fold increased risk of significant aortic regurgitation. Assessing the spatial distribution of calcification using CT angiography helps in planning the procedure and improving procedural outcomes [3].

Different TAVR platforms have varying capabilities for handling severe calcification. Extrapolating from data regarding bicuspid aortic valves, use of a balloon-expandable valve (BEV) is associated with a lower risk of PVL, though a higher risk of annular rupture. Conversely, the risk of annular rupture is lower with a self-expandable valve (SEV), but the risk of PVL is higher [5-7]. Several studies and meta-analyses have highlighted a link between moderate or severe PVL and increased morbidity and mortality following TAVR [8,9]. Our choice of BEV in this case was due to the high likelihood of PVL following TAVR, regardless of valve platform choice. Furthermore, in our experience, PVL closure is somewhat more straightforward in the setting of a BEV versus an SEV because the nitinol frame of an SEV exhibits gradual radial force and conformability, resulting in a dynamic annular interface that complicates precise device deployment.

In cases of significant PVL due to severe aortic annular calcification, percutaneous PVL closure can be utilized as an alternative to aggressive post-dilation to mitigate the risk of annular rupture. The Amplatzer Vascular Plug II device has proven effective in accommodating the complex, crescentic, and serpentine anatomy often associated with aortic PVL, providing efficient sealing around calcific nodules [10]. Other devices, such as the Amplatzer Vascular Plug I, III, and IV, and the Ductal Occluder II (Abbott), can be considered, depending on anatomical considerations. Clinical reports indicate that aortic PVL closure effectively addresses significant regurgitation around balloon-expandable TAVR valves, leading to symptom relief and improved patient outcomes [11].

## Conclusions

Severe aortic annular calcification impacts TAVR outcomes, including increasing the risk of PVL and annular rupture. Careful pre-procedural assessment and TAVR device selection are crucial to minimizing complications. PVL closure is a safe alternative to aggressive post-dilation to treat PVL in such cases.
